# Best-Evidence Systematic Review and Meta-Analysis of Mini-Open Carpal Tunnel Release

**DOI:** 10.1016/j.jhsg.2023.08.005

**Published:** 2023-09-27

**Authors:** Warren C. Hammert, Kevin C. Chung, Larry E. Miller

**Affiliations:** ∗ Department of Orthopaedic Surgery, Division of Hand Surgery, Duke University Medical Center, Durham, NC; † University of Michigan Comprehensive Hand Center, Michigan Medicine, Ann Arbor, MI; ‡ Miller Scientific, Johnson City, TN

**Keywords:** Best evidence, Carpal tunnel release, Carpal tunnel syndrome, Meta-analysis, Mini-open, Systematic review

## Abstract

**Purpose:**

This study aimed to evaluate the safety and effectiveness of mini-open carpal tunnel release (mOCTR) using best-evidence synthesis methods.

**Methods:**

We systematically searched for prospective studies published from January 2013 to July 2023 that reported outcomes from a minimum of 50 mOCTR cases. The outcomes included Quick Disabilities of the Arm, Shoulder, and Hand Questionnaire, Boston Carpal Tunnel Questionnaire Symptom Severity Scale (BCTQ-SSS) and Functional Status Scale (BCTQ-FSS), pain visual analog scale (VAS), complication rate, and reoperation rate. Data analysis was performed using a random-effects meta-analysis, with metaregression to identify the associations between patient- and study-level factors with surgical outcomes.

**Results:**

The meta-analysis included 23 studies with 2,303 patients followed for median durations ranging from 6 to 12 months depending on the outcome. Mini-open carpal tunnel release resulted in statistically significant and clinically important improvements in Quick Disabilities of the Arm, Shoulder, and Hand Questionnaire (mean difference = −25.5; 95% confidence interval [CI]: −36.4 to −14.5; *P* < .001), BCTQ-SSS (mean difference = −2.2; 95% CI: −2.5 to −1.9; *P* < .001), BCTQ-FSS (mean difference = −2.1; 95% CI: −2.4 to −1.7; *P* < .001), and pain VAS (mean difference = −5.1; 95% CI: −6.2 to −4.1; *P* < .001). The sole predictor of improvement in BCTQ-SSS, BCTQ-FSS, and pain VAS was a higher preoperative score for the respective variable (all *P* < .001). The risk of complications (mainly short-term pillar pain or scar complications) was 8.9% (95% CI: 4.0%–13.8%) and increased with longer incision lengths (*P* = .008). Revision carpal tunnel release was performed in 0.6% (95% CI: 0.1%–1.0%) of the cases during follow-up. No cases of median nerve transection were reported.

**Conclusions:**

Based on a best-evidence meta-analysis of contemporary studies, mOCTR significantly improved function and pain, with a relatively low risk of mainly temporary complications. Patient outcomes after mOCTR were influenced by patient symptomatology and surgical incision length.

**Clinical relevance:**

Mini-open carpal tunnel release is an effective surgical option that significantly improves symptoms and function, especially for patients with more severe baseline dysfunction. Surgeons should use the shortest incision that allows adequate visualization to safely divide the transverse carpal ligament.

Carpal tunnel syndrome (CTS) is a prevalent neuropathic disorder affecting approximately 5% of adults.^1^ Carpal tunnel syndrome is characterized by paresthesia, numbness, and weakness secondary to the entrapment of the median nerve within the carpal tunnel. Available treatment options range from conservative strategies such as splinting and corticosteroid injections to more invasive surgical interventions. If nonoperative treatments are unsuccessful, the definitive treatment is carpal tunnel release (CTR).^2^ Approximately 600,000 CTR procedures are performed annually in the United States alone.^1^^,^^3^ Magnetic resonance imaging studies have confirmed that division of the transverse carpal ligament during CTR increases carpal tunnel volume by 24% and shifts the median nerve anteriorly by 3.5 mm,^4^^,^^5^ thus alleviating compressive symptoms. Various CTR techniques are available to the physician to access the transverse carpal ligament including open CTR (OCTR), mini-open CTR (mOCTR), endoscopic CTR (ECTR), or ultrasound-guided CTR. A recent trend toward smaller incisions to minimize surgical trauma and postoperative pain and improve appearance has been observed.^6^, ^7^, ^8^, ^9^, ^10^ Mini-open CTR is the most commonly used CTR technique in the United States,^8^ combining the visualization advantages of OCTR with a less invasive surgical approach.

Several meta-analyses have reported clinical outcomes of OCTR^11^, ^12^, ^13^, ^14^, ^15^, ^16^, ^17^; however, few have specifically evaluated mOCTR.^18^^,^^19^ Although meta-analyses of randomized controlled trials (RCTs) are considered the standard for comparing the safety and effectiveness of different therapies, they provide limited insights into the expected results from a single technique. For example, a comparative meta-analysis may conclude that treatment A carries a higher risk of reoperation than treatment B based on a risk ratio of 1.5. However, without additional context, it is unclear whether the actual reoperation rates in each group were 1.5% versus 1.0%, 15% versus 10%, or even 75% versus 50%. Furthermore, most medical literature comprises observational studies, which are ignored in the meta-analyses of RCTs. For instance, a MEDLINE search by the authors revealed that only 6% of the studies published in the past decade with CTR in the title were classified as an RCT. To overcome these challenges, meta-analyses can integrate data from RCTs and high-quality observational studies. This inclusive approach facilitates reporting results for individual interventions derived from a more diverse body of literature, thereby improving the generalizability of the findings. Such methodology is further justified because the outcomes in individual treatment groups tend to be similar in RCTs and observational studies.^20^^,^^21^ Thus, the objective of this systematic review and meta-analysis was to summarize the safety and effectiveness outcomes from contemporary mOCTR literature using best-evidence principles to select the highest-quality studies.

## Methods

This systematic review and meta-analysis followed the best-evidence synthesis methods proposed by Slavin^22^ and the recommendations outlined in the Preferred Reporting Items for Systematic Reviews and Meta-analyses.^23^ The review protocol was prospectively registered at www.researchregistry.com (reviewregistry1664).

### Study eligibility criteria

Eligible studies used prospective patient enrollment, treated a minimum of 50 hands using mOCTR or a limited incision CTR technique, reported at least one outcome specified in this review within a 5-year postoperative follow-up period, and were published between January 2013 and June 2023 without language restrictions. We excluded the studies reporting results from various CTR techniques, studies of patients treated exclusively with concurrent procedures or revision CTR, studies using endoscopy or ultrasound for enhanced visualization, and studies published as abstracts or duplicate publications. The rationale for the best-evidence criteria is provided in Supplementary Table 1, available online on the Journal’s website at https://www.jhsgo.org.

### Search strategy and study selection process

A systematic search was performed using MEDLINE, Embase, and the Cochrane Central Register of Controlled Trials. Anticipating the challenge of correctly classifying a procedure as mOCTR based only on the title and abstract, we developed a broad search strategy to maximize sensitivity. For MEDLINE searches, we used the diagnosis terms [carpal tunnel, median neuropath∗, and median nerve] combined with procedure terms [decompression, incision, limited, mini-open, open, release, and surg∗], a strategy intended to retrieve all CTR studies irrespective of the technique. We adapted these search strategies as needed for the other databases. Supplementary manual searches were also performed in the Directory of Open Access Journals, Google Scholar, and the reference lists of eligible studies and review articles. Two researchers (L. Miller and D. Gains) independently screened articles for eligibility, and the full texts of all potentially relevant studies were subsequently reviewed. We resolved discrepancies in study eligibility through discussion and consensus. The final search was conducted in July 2023.

### Data extraction and outcomes

The researchers independently extracted data from eligible studies using standardized data collection forms, with discrepancies resolved through discussion and consensus. The methodological quality of the studies was evaluated using the National Institutes of Health assessment tool for before-after studies.^24^ The main outcomes of this meta-analysis included the Quick Disabilities of the Arm, Shoulder, and Hand Questionnaire (*Quick*DASH), Boston Carpal Tunnel Questionnaire Symptom Severity Scale (BCTQ-SSS) and Functional Status Scale (BCTQ-FSS) scores, pain visual analog scale (VAS), complications, and reoperations. The *Quick*DASH was analyzed on a 0–100 scale, the BCTQ on a 1–5 scale, and pain VAS on a 0–10 scale. For studies reporting these outcomes at multiple time points, we included the values at the final follow-up visit in the analysis. The minimal clinically important differences (MCIDs) for postoperative changes in patient-reported outcomes (PROs) were −15 points for *Quick*DASH,^25^ −1.14 points for BCTQ-SSS,^26^ −0.74 points for BCTQ-FSS,^26^ and −2 points for pain VAS.^27^ We evaluated complications and reoperations based on all events reported during the postoperative follow-up period.

### Statistical methods

We analyzed continuous outcomes using the mean difference from baseline and 95% confidence interval (CI) for individual studies and the overall estimate. We reported complications and reoperations using the event rate and 95% CI. A restricted maximum likelihood random-effects meta-analysis was used to calculate the overall results, accounting for heterogeneity among studies. For studies that reported outcomes from multiple mOCTR subgroups, we combined the data to form a single mOCTR group for each outcome in each study.^28^ We estimated the heterogeneity of outcomes among the studies with the *I*^2^ statistic, with 0% indicating no heterogeneity and larger values indicating increasing heterogeneity.^29^ We assessed potential publication bias visually through funnel plot symmetry and using the trim-and-fill method, which recalculated the meta-analysis results based on the estimated number of studies missing due to publication bias.^30^ A one-study-removed meta-analysis was conducted to evaluate the influence of single-study effects on outcomes. We used metaregression to examine the association of patient- and study-level factors with mOCTR outcomes reported in at least five studies and with significant observed heterogeneity (*I*^2^ > 50%). The independent variables of interest included study sample size, age, sex, preoperative value for PROs, duration of symptoms, surgical incision length, and follow-up duration.

## Results

### Patient and study characteristics

From the 506 titles and abstracts identified in the searches, we fully reviewed 106 articles where the primary reasons for exclusion were retrospective study design (30), insufficient sample size (29), or the lack of information on the specific CTR technique (12). The meta-analysis included 23 studies,^31^, ^32^, ^33^, ^34^, ^35^, ^36^, ^37^, ^38^, ^39^, ^40^, ^41^, ^42^, ^43^, ^44^, ^45^, ^46^, ^47^, ^48^, ^49^, ^50^, ^51^, ^52^, ^53^ involving 2,303 patients from 15 countries, representing a best-evidence synthesis of mOCTR literature published over the past decade (Supplementary Figure 1). The median age of participants was 55 years (study-wide range was 35–63 years), most were women (median 74%; study-wide range was 50%–100%), and the median CTS symptom duration before surgery was 12 months (study-wide range was 5–57 months; Table 1). Before surgery, patient-reported outcome values were 40.3 (95% CI: 28.5–52.1) for Q-DASH, 3.5 (95% CI: 3.3–3.7) for BCTQ-SSS, 3.4 (95% CI: 3.1–3.7) for BCTQ-FSS, and 5.9 (95% CI: 4.6–7.2) for pain VAS. The methodological quality of studies was rated good for 15 studies and fair for 8 studies; none were rated poor (Supplementary Table 2).

**Table 1:** Characteristics of Best-Evidence Studies of mOCTR

Study	Surgery Dates	Country	Patients	Hands	Age (y)	Female Sex (%)	CTS duration (mo)	CTR Incision Location
Al-Sudani (2015)^31^	2003–2014	Iran	94	113	35	98	12	1 cm longitudinal incision in proximal palm
Carmo (2019)^32^	2008–2016	Portugal	116	157	55	85	9	1 cm transverse wrist incision
Chen (2017)^33^	2010–2012	China	49	55	47	69	—	Double incisions: 1 cm longitudinal incision distal to the intersection of the third web axis and thumb axis, and 1.5 cm transverse incision at wrist crease
Cho (2016)^34^	2010–2012	Korea	79	79	54	92	—	2 cm longitudinal incision ulnar to the palmar crease, or 1.5 cm transverse incision at the distal wrist crease
Fazil (2022)^35^	2017–2019	India	122	122	45	74	5	0.5 cm longitudinal incision at base of wrist, or 1.5–2 cm transverse incision at the distal wrist crease
Gil (2020)^36^	—	US	67	—	55	69	34	1.5–2 cm longitudinal incision in the mid-palm
Gulabi (2014)^37^	1997–2003	Turkey	69	69	55	67	—	3–3.5 cm palm-only mini-incision between distal wrist crease and Kaplan’s cardinal line
Kalhoro (2021)^38^	2012–2020	Pakistan	97	97	40	89	—	1 cm transverse incision at the transverse wrist crease
Korkmaz (2013)^39^	2007–2009	Turkey	79	93	54	81	—	2–3 cm longitudinal incision ulnar to the palmar longus, or 2 cm transverse incision at the distal palmar crease
Ma (2021)^40^	2017–2020	China	85	85	49	73	19	0.5 cm transverse incision along the proximal wrist
Mardanpour (2018)^41^	2011–2015	Iran	188	300	40	70	—	1.5 cm longitudinal incision starting 2 cm to the distal flexure wrist crease
Martinez-Catasús (2018)^42^	2009–2010	Spain	52	52	58	75	5	<1 cm longitudinal incision between thenar and hypothenar eminences
Ozer (2013)^43^	2007–2011	US	114	114	48	69	—	1 cm longitudinal incision at the proximal palm
Ramos-Zúñiga (2017)^44^	—	Mexico	55	65	—	100	—	1.5 cm incision in the thenar sulcus
Ranjeet (2022)^45^	2019–2020	Nepal	78	86	56	77	—	2–3 cm curvilinear incision beginning proximal to Kaplan’s line and extending to volar flexion crease of wrist
Saaiq (2021)^46^	2016–2020	Iran	67	77	41	81	9	2–2.5 cm curvilinear incision, 6–10 mm ulnar to the thenar crease
Suwannaphisit (2021)^47^	2018–2020	Thailand	142	142	59	85	16	1.5 cm longitudinal incision in line with the radial half of the ring finger and not crossing the wrist flexor crease
Tarallo (2014)^48^	2009–2011	Italy	60	60	63	50	—	2 cm longitudinal incision in the proximal palm
van den Broeke (2019)^49^	2015–2016	Netherlands	72	72	58	74	12	1–2 cm longitudinal incision to the distal wrist crease
Vanni (2015)^50^	2011–	Italy	110	119	56	74	—	0.6 cm longitudinal incision distal to the proximal wrist, along the line from the third finger web space to the wrist
Zhang (2016)^51^	2008–2010	China	73	73	46	68	6	Double incisions: 1 cm transverse incision 3 mm proximal to the middle flexor wrist crease, and 1.5 cm longitudinal incision 1 cm distal to the distal wrist crease
Zhang (2023)^52^	2019–2021	US	63	63	60	70	—	1.5–2 cm longitudinal incision from ulnar aspect of the distal palmaris longus tendon to the third webspace, just proximal to Kaplan’s cardinal line
Zyluk (2020)^53^	2016–2017	Poland	372	372	57	82	57	2 cm longitudinal incision in the proximal palm, extending to the distal wrist crease

### mOCTR effectiveness

**Figure 1:**
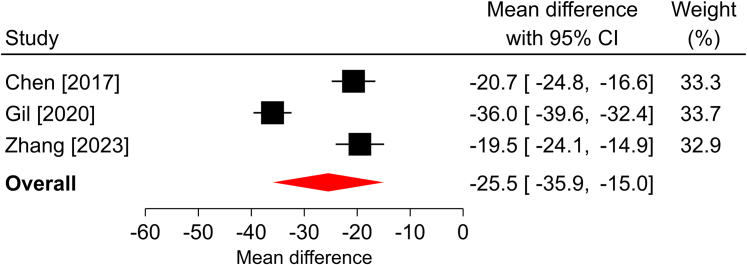
Forest plot of change in *Quick*DASH after mOCTR. The mean difference from baseline and 95% CI are plotted for each study. The size of the square is proportional to the weighting of the study in the meta-analysis. The overall mean difference is denoted by the diamond apex, and the 95% CI is denoted by the diamond width. The overall mean difference was −25.5 (*P* < .001) over a median 6-month follow-up. Significant heterogeneity (*I*^2^=95%) was identified among the studies.

**Figure 2:**
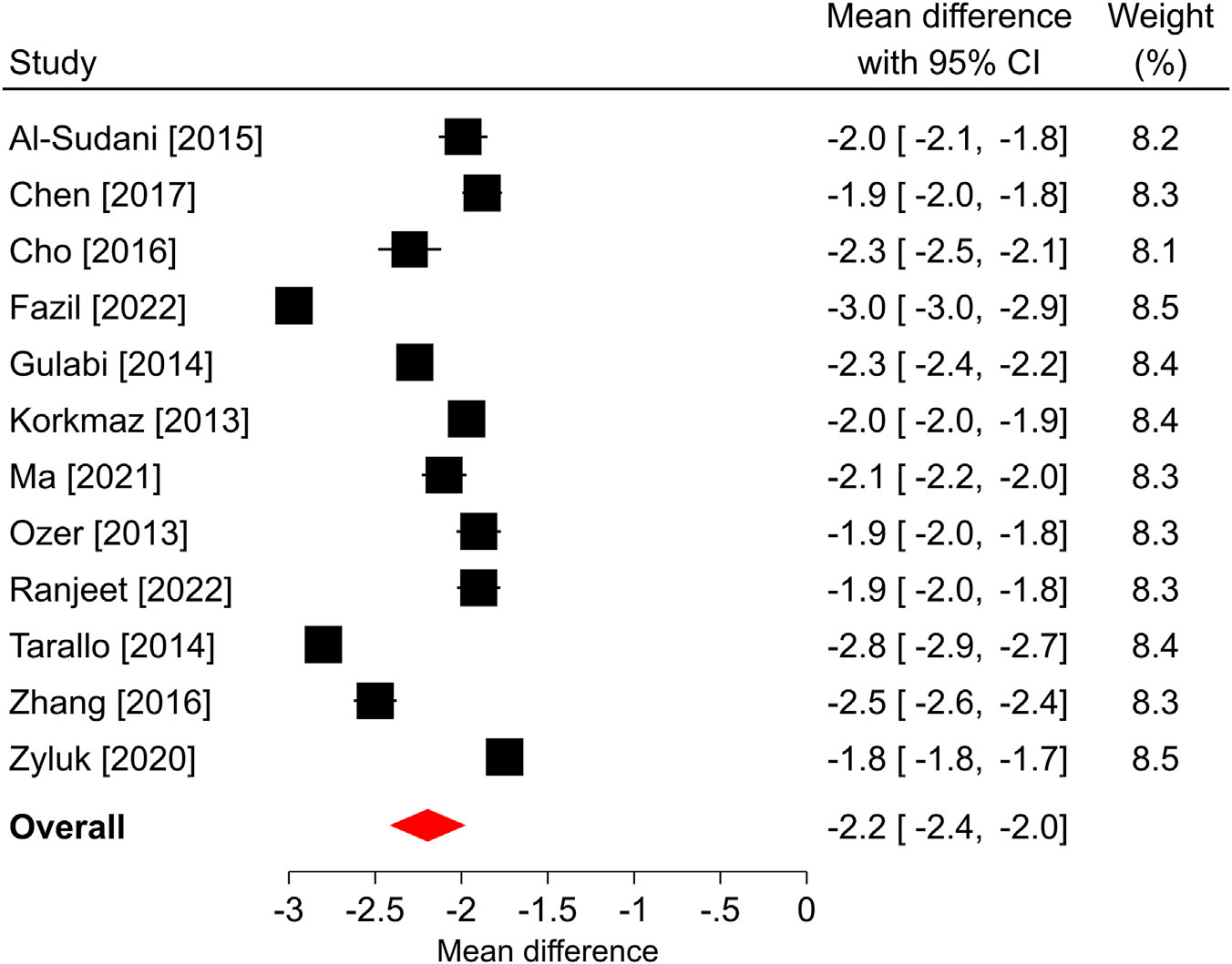
Forest plot of change in BCTQ-SSS after mOCTR. The mean difference from baseline and 95% CI are plotted for each study. The size of the square is proportional to the weighting of the study in the meta-analysis. The overall mean difference is denoted by the diamond apex, and the 95% CI is denoted by the diamond width. The overall mean difference was −2.2 (*P* < .001) over a median 6-month follow-up. Significant heterogeneity (*I*^2^ > 99%) was identified among the studies.

**Figure 3:**
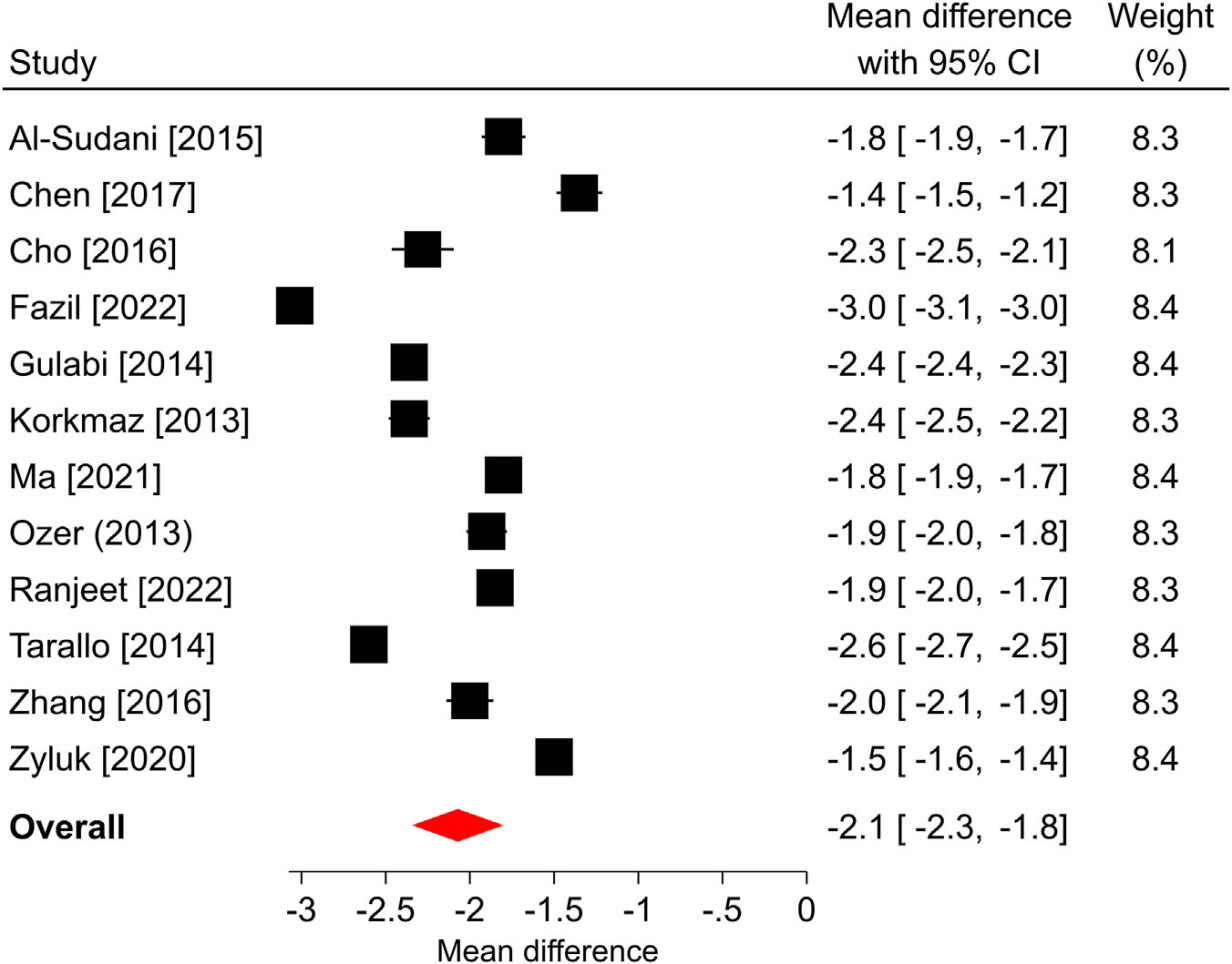
Forest plot of change in BCTQ-FSS after mOCTR. The mean difference from baseline and 95% CI are plotted for each study. The size of the square is proportional to the weighting of the study in the meta-analysis. The overall mean difference is denoted by the diamond apex, and the 95% CI is denoted by the diamond width. The overall mean difference was −2.1 (*P* < .001) over a median 6-month follow-up. Significant heterogeneity (*I*^2^ = 99%) was identified among the studies.

**Figure 4:**
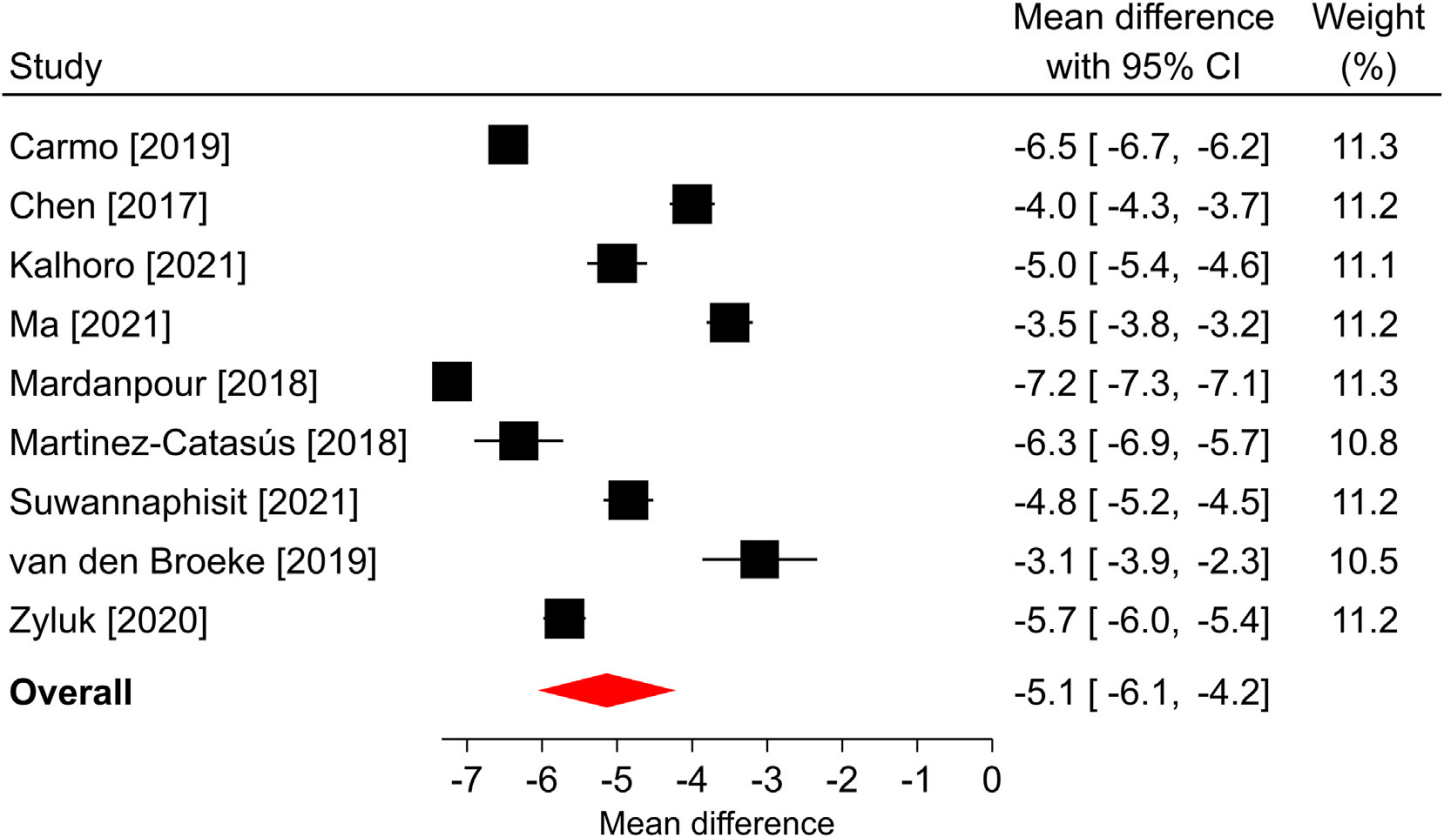
Forest plot of change in pain VAS after mOCTR. The mean difference from baseline and 95% CI are plotted for each study. The size of the square is proportional to the weighting of the study in the meta-analysis. The overall mean difference is denoted by the diamond apex, and the 95% CI is denoted by the diamond width. The overall mean difference was −5.1 (*P* < .001) over a median 6-month follow-up. Significant heterogeneity (*I*^2^ = 99%) was identified among the studies.

In the three studies that reported a change in *Quick*DASH scores over a median follow-up of 6 months, all reported statistically significant improvements compared with the preoperative scores. The overall mean difference was −25.5 points (95% CI: −36.4 to −14.5; *P* < .001), and significant heterogeneity was found among the studies (*I*^2^ = 95%; Fig. 1). The BCTQ-SSS scores statistically improved in each of the 12 studies reporting this outcome and in the overall analysis over a median of 6-month follow-up. The overall mean difference was −2.2 points (95% CI: −2.5 to −1.9; *P* < .001), with significant heterogeneity identified among the studies (*I*^2^ > 99%; Fig. 2). Similar findings were observed in the 12 studies reporting BCTQ-FSS, where all reported statistically significant improvements over a median of 6-month follow-up, the overall mean difference was −2.1 points (95% CI: −2.4 to −1.7; *P* < .001), and significant heterogeneity was observed (*I*^2^ = 99%; Fig. 3). Among the nine studies reporting a change in pain VAS over a median of 6-month follow-up, all reported statistically significant improvements compared with the preoperative scores. The overall mean difference was −5.1 points (95% CI: −6.2 to −4.1; *P* < .001), with significant heterogeneity identified among studies (*I*^2^ = 99%; Fig. 4). The overall mean difference and the entire 95% CI exceeded the MCID for each of these outcomes, indicating that the improvements after surgery were clinically important.

## mOCTR complications

Postsurgical complications were reported in 8.9% (95% CI: 4.0%–13.8%) of the cases over a median follow-up of 12 months, and significant heterogeneity was observed among the studies (*I*^2^ = 98%; Supplementary Figure 2). Among the 133 total complications reported in the studies, the most common were pillar pain (44), scar tenderness/sensitivity (35), temporary nerve irritation (10), superficial wound infection (7), delayed wound healing (7), and excessive bleeding (7). No median nerve transections or deep wound infections were reported. Reoperations were performed in 0.6% (95% CI: 0.1%–1.0%) of the cases over a median follow-up of 9 months (Supplementary Figure 3), and heterogeneity among studies was negligible (*I*^2^ = 0%).

### Meta-analysis diagnostics

The trim-and-fill analysis yielded identical estimates to the main analysis for all outcomes, except complications, which were 8.9% in the main analysis and 10.1% in the trim-and-fill analysis. This suggests that publication bias was negligible for all outcomes and that the meta-analysis results were not affected by selective publication based on the direction or strength of the study findings.

No single study significantly influenced the overall conclusions of the meta-analysis. When re-running the meta-analysis excluding each study one at a time, the overall mean differences ranged from −28.4 to −20.2 for *Quick*DASH (all *P* < .001), −2.2 to −2.1 for BCTQ-SSS (all *P* < .001), −2.1 to −2.0 for BCTQ-FSS (all *P* < .001), and −5.4 to −4.9 for pain VAS (all *P* < .001). As in the primary analysis, the overall mean difference and the entire 95% CI in the one-study-removed sensitivity analysis exceeded the MCID for each outcome. Similarly, the overall event rates were minimally influenced in the one-study-removed sensitivity analyses with values ranging from 6.5% to 9.5% for complications and 0.5% to 0.6% for reoperations.

Four outcomes met the criteria for inclusion in the metaregression based on the number of studies reporting the outcome and the presence of significant heterogeneity as follows: BCTQ-SSS, BCTQ-FSS, pain VAS, and complication rate (Table 2). The baseline value was the strongest predictor of postoperative change in BCTQ-SSS, BCTQ-FSS, and pain VAS, where more severe preoperative symptoms were associated with greater postoperative improvement (all *P* < .001; Supplementary Figures 4–6). A longer incision length was associated with an increased risk of postoperative complications (*P* = .008; Supplementary Figure 7).

**Table 2:** Metaregression of Patient- and Study-Factors on Outcomes Following mOCTR∗

Variable	BCTQ-SSS Mean Difference†	BCTQ-FSS Mean Difference†	Pain VAS Mean Difference†	Complications‡
Baseline score	**z = –6.42** ***P* < .001**	**z = −7.13** ***P* < .001**	**z = –4.48** ***P* < .001**	**—**
CTS duration (mo)	z = 1.91 *P* = .06	z = 1.41 *P* = .16	z = −0.11 *P* = .91	z = −1.75 *P* = .08
Follow-up (mo)	z = −1.85 *P* = .06	z = −0.62 *P* = .53	z = -0.37 *P* = .71	z = −0.64 *P* = .53
Female (%)	z = 1.50 *P* = .13	z = 0.78 *P* = .44	z = −0.46 *P* = .65	z = −1.96 *P* = .05
Sample size	z = 1.17 *P* = .24	z = 1.00 *P* = .32	z = −1.79 *P* = .07	z = −0.97 *P* = .33
Age (y)	z = −0.24 *P* = .81	z = −0.60 *P* = .55	z = 0.40 *P* = .69	z = −0.28 *P* = .78
Incision length (cm)	z = 0.19 *P* = .85	z = 0.11 *P* = .91	z = 0.15 *P* = .88	**z = 2.64** ***P* = .008**

∗ Statistically significant associations in bold.

† Negative z-scores indicate that as the value for the baseline variable increases, the mean difference for the outcome decreases (ie, improves).

‡ Negative z-scores indicate that as the value for the baseline variable increases, the complication rate decreases.

## Discussion

This systematic review and meta-analysis evaluated 23 contemporary prospective mOCTR studies, applying best-evidence principles to select the most methodologically rigorous studies. The principal finding of the meta-analysis was a statistically significant and clinically important improvement in postoperative function and symptoms after mOCTR. Second, the extent of improvement in PROs was directly correlated with the severity of preoperative symptoms, suggesting that patients with more severe symptoms demonstrated the greatest absolute improvement. Third, the overall complication rate was 8.9%, which was shown to correlate with surgical incision length. Fourth, the outcomes reported in this meta-analysis were reported over relatively short follow-up periods, ranging from 6 to 12 months. Finally, the majority of recent mOCTR literature consists of studies of lower methodological quality, as indicated by the large number of studies excluded from this review due to retrospective designs and small sample sizes. Overall, based on the best evidence in the literature, short-term outcomes with mOCTR appear favorable and are influenced by patient symptomatology and surgical technique.

An indirect comparison of the results from this best-evidence review of mOCTR literature with a recent best-evidence review of ECTR literature^54^ yields some interesting findings. The postoperative improvements in *Quick*DASH (−25.5 points with mOCTR; −28.8 points with ECTR) and pain VAS (−5.1 points with mOCTR; −5.1 points with ECTR) were generally comparable. Although the absolute improvement in BCTQ-SSS (−2.2 points with mOCTR; −1.8 points with ECTR) and BCTQ-FSS (−2.1 points with mOCTR; −1.5 points with ECTR) favored mOCTR, these advantages were negated when accounting for preoperative status. This is because mOCTR patients had higher preoperative scores (3.5 vs 3.1 for BCTQ-SSS; 3.4 vs 2.9 for BCTQ-FSS), and both reviews demonstrated a relationship between higher preoperative scores and larger postoperative changes. Overall, once differences in baseline symptoms are accounted for, PROs are generally comparable with mOCTR and ECTR.

One notable divergence between these best-evidence reviews pertained to complication rates. The complication rate was 0.7% in the ECTR review,^54^ but 8.9% in the current mOCTR review. A plausible explanation for this difference relates to the surgical incision length. The current review noted a relationship between longer incision lengths and higher rates of postoperative complications, most commonly pillar pain or scar complications. Longer incisions may result in greater disruption of local soft tissues, including cutaneous nerves, fascial structures, and vascular supply, leading to increased postoperative inflammation, scarring, and potential nerve hypersensitivity. Although the duration of postoperative complications was reported inconsistently in the included mOCTR studies, most cases of pillar pain and scar sensitivity resolved during the follow-up period. Reoperation rates were low with mOCTR (0.6%) and were comparable with those reported in the best-evidence ECTR review (0.5%).^54^ Thus, it appears that mOCTR and ECTR yield comparable patient outcomes, with the exception of temporary pain or scar-related complications associated with longer incision lengths in mOCTR.

This review is subject to several limitations. First, notable inconsistency exists in the classification and reporting of mOCTR techniques across different studies. For example, some studies described the procedure as OCTR in the title or abstract but described a limited incision or mOCTR procedure within the main text. Furthermore, many studies were excluded from this review because they provided no surgical procedure details. This raises the possibility that some studies employing mOCTR techniques may have been excluded from this review due to insufficient procedural descriptions. Second, considerable variation exists in the mOCTR techniques used in these studies, including longitudinal, transverse, or dual incisions, each initiating at slightly different anatomical locations, and with incision lengths ranging from 0.5 to 3.5 cm. Thus, we recommend standardizing the reporting of CTR techniques based on important parameters such as incision location, incision length, and use of assistive visualization tools instead of the generally nonspecific nomenclature frequently used in the literature. Finally, this review did not make comparisons to other CTR techniques due to an insufficient number of high-quality prospective comparative studies. As a result, drawing conclusions from this meta-analysis based on comparisons with other CTR studies should be made cautiously.

## Conclusions

Based on a best-evidence meta-analysis of contemporary studies, mOCTR significantly improved function and pain, with a relatively low risk of mainly temporary complications. Patient outcomes after mOCTR were influenced by patient symptomatology and surgical incision length.
