# Supplementary material for: Best-Evidence Systematic Review and Meta-Analysis of Mini-Open Carpal Tunnel Release
Source: J Hand Surg Glob Online. 2023 Sep 27;6(1):35–42. doi: 10.1016/j.jhsg.2023.08.005 (PMC10837294; doi:10.1016/j.jhsg.2023.08.005)
Supplement: Supplementary Tables and Figures [file mmc1.docx]

**Supplement**

**Supplement Table 1**

Best-evidence Study Eligibility Criteria

| **Criterion** | **Rationale** |
| --- | --- |
| ***Inclusion*** |  |
| Prospective study | Minimizes risk of selection bias |
| Minimum 50 hands treated | Inclusion of larger studies reduces small-study effects such as selection bias, publication bias, and inflated effect sizes, thereby enhancing precision and generalizability of the meta-analysis findings |
| Reported at least one outcome | Necessary to develop meta-analysis estimates |
| No language restrictions | Reduces risk of language bias |
| Publication between 2013 and 2023 | Ensures that the meta-analysis incorporates the most current and relevant evidence |
| ***Exclusion*** |  |
| Various or unclear CTR technique | Ensures strict focus on outcomes with mOCTR only |
| Concomitant surgical procedures | Minimizes confounding of results due to effects of the concomitant procedure |
| Revision CTR study | Patient outcomes are inferior in revision vs. primary CTR |
| Published as abstract only | Abstracts lack sufficient detail with which to evaluate methodological quality and determine the risk of bias accurately |
| Duplicate publication | Avoids duplication of data from common patients |

CTR, carpal tunnel release; mOCTR, mini-open carpal tunnel release.

**Supplement Table 2**

Methodological Study Quality Assessment using the National Institute of Health Assessment Tool for Before-After Studies.^*^

| **Study** | **1** | **2** | **3** | **4** | **5** | **6** | **7** | **8** | **9** | **10** | **11** | **12** | **Quality**  **Rating** |
| --- | --- | --- | --- | --- | --- | --- | --- | --- | --- | --- | --- | --- | --- |
| Al-Sudani [2015] ^31^ | Y | N | Y | Y | Y | Y | Y | N | N | N | N | ^†^ | Fair |
| Carmo [2019] ^32^ | Y | Y | Y | Y | Y | Y | N | N | N | N | N | ^†^ | Fair |
| Chen [2017] ^33^ | Y | Y | Y | Y | Y | Y | Y | N | N | Y | N | ^†^ | Good |
| Cho [2016] ^34^ | Y | Y | Y | Y | Y | Y | Y | N | Y | Y | N | ^†^ | Good |
| Fazil [2022] ^35^ | Y | Y | Y | Y | Y | Y | Y | N | N | Y | N | ^†^ | Good |
| Gil [2020] ^36^ | Y | Y | Y | Y | Y | Y | Y | N | Y | Y | N | ^†^ | Good |
| Gulabi [2014] ^37^ | Y | Y | Y | Y | Y | Y | Y | N | Y | Y | N | ^†^ | Good |
| Kalhoro [2021] ^38^ | N | Y | Y | Y | Y | Y | Y | N | N | N | N | ^†^ | Fair |
| Korkmaz [2013] ^39^ | Y | Y | Y | Y | Y | Y | Y | N | N | Y | N | ^†^ | Good |
| Ma [2021] ^40^ | Y | Y | Y | Y | Y | Y | Y | N | Y | Y | N | ^†^ | Good |
| Mardanpour [2018] ^41^ | Y | Y | Y | Y | Y | Y | Y | N | N | Y | N | ^†^ | Good |
| Martinez-Catasús [2018] ^42^ | Y | Y | Y | Y | Y | Y | Y | N | N | Y | N | ^†^ | Good |
| Ozer [2013] ^43^ | Y | N | Y | Y | Y | Y | Y | N | N | N | N | ^†^ | Fair |
| Ramos-Zúñiga [2017] ^44^ | Y | Y | Y | Y | Y | Y | N | N | N | N | N | ^†^ | Fair |
| Ranjeet [2022] ^45^ | Y | Y | Y | Y | Y | Y | Y | N | Y | N | N | ^†^ | Good |
| Saaiq [2021] ^46^ | Y | Y | Y | Y | Y | Y | Y | N | N | N | N | ^†^ | Fair |
| Suwannaphisit [2021] ^47^ | Y | Y | Y | Y | Y | Y | Y | Y | Y | Y | N | ^†^ | Good |
| Tarallo [2014] ^48^ | Y | Y | Y | Y | Y | Y | Y | N | N | Y | N | ^†^ | Good |
| van den Broeke [2019] ^49^ | Y | Y | Y | Y | Y | Y | Y | N | Y | Y | N | ^†^ | Good |
| Vanni [2015] ^50^ | Y | Y | Y | Y | Y | Y | Y | Y | N | N | N | ^†^ | Good |
| Zhang [2016] ^51^ | Y | Y | Y | Y | Y | Y | Y | N | N | N | N | ^†^ | Fair |
| Zhang [2023] ^52^ | Y | Y | Y | Y | Y | Y | Y | N | N | N | N | ^†^ | Fair |
| Zyluk [2020] ^53^ | Y | Y | Y | Y | Y | Y | Y | N | N | Y | N | ^†^ | Good |

^*^Item numbers and associated descriptions include: 1) objective clearly stated; (2) eligibility criteria described; (3) representative patient population; (4) all eligible participants enrolled in study; (5) sufficient sample size; (6) intervention described; (7) outcome measures specified; (8) outcome assessors blinded; (9) loss to follow-up and intention-to-treat analysis; (10) statistical analysis of outcome measures before and after intervention; (11) interrupted time-series design; (12) individual data used for group-level effects.

^†^Not applicable.


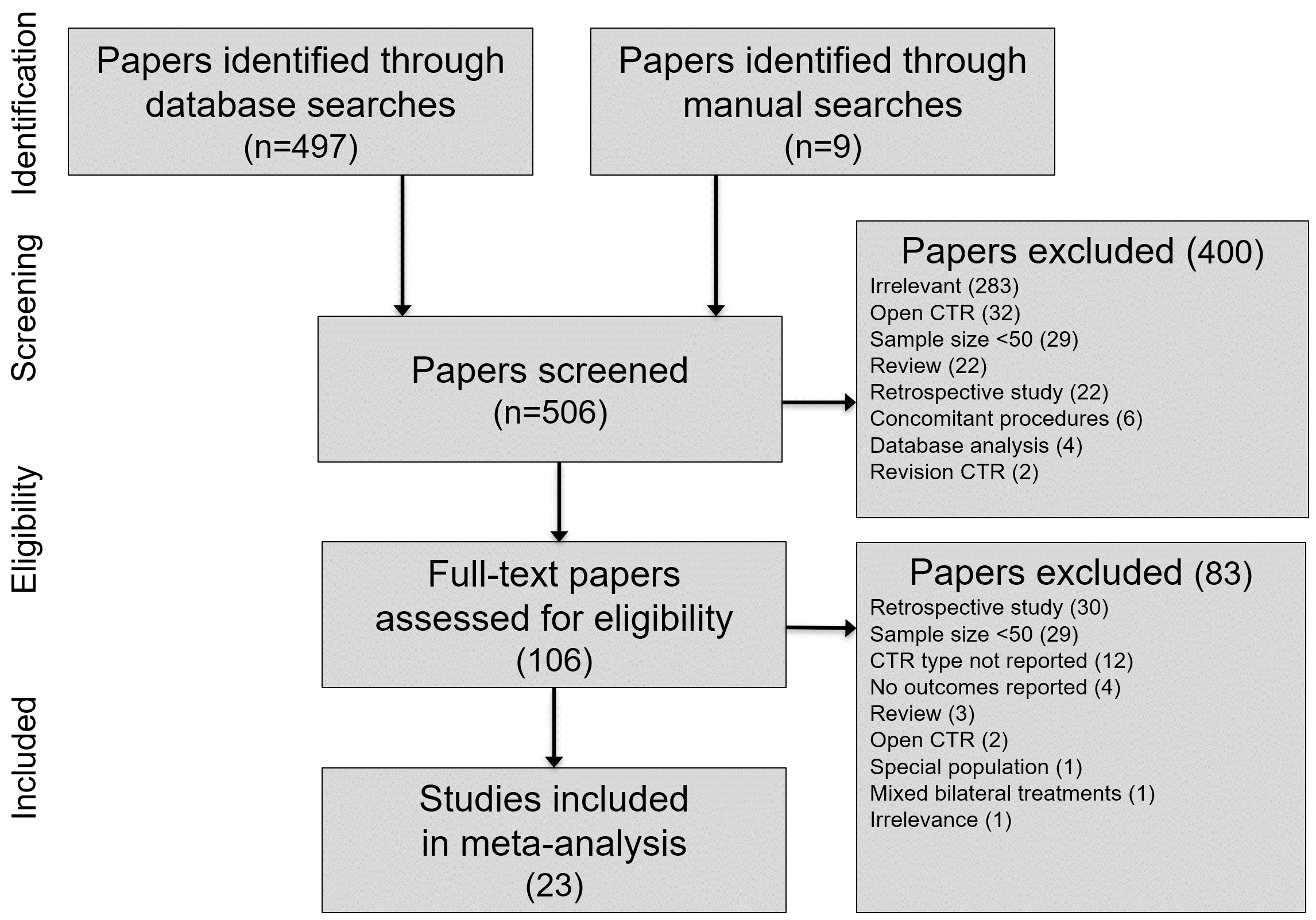


**Supplement Figure 1.** PRISMA flow diagram. CTR, carpal tunnel release.

**Supplement Figure 2.** Forest plot of complications after mini-open carpal tunnel release. The proportion and 95% confidence interval are plotted for each study. The size of the square is proportional to the weighting of the study in the meta-analysis. The overall proportion is denoted by the diamond apex and the 95% confidence interval is denoted by the diamond width. The overall event rate was 8.9% over a median 12-month follow-up. Significant heterogeneity (*I*^2^=98%) was identified among studies. The proportions for studies with no events may exceed zero in a random-effects meta-analysis model due to shrinkage towards the pooled estimate.

**Supplement Figure 3.** Forest plot of reoperations after mini-open carpal tunnel release. The proportion and 95% confidence interval are plotted for each study. The size of the square is proportional to the weighting of the study in the meta-analysis. The overall proportion is denoted by the diamond apex and the 95% confidence interval is denoted by the diamond width. The overall event rate was 0.6% over a median 9-month follow-up. Negligible heterogeneity (*I*^2^=0%) was identified among studies. The proportions for studies with no events may exceed zero in a random-effects meta-analysis model due to shrinkage towards the pooled estimate.

**Supplement Figure 4.** Bubble plot of the association between the change in BCTQ-SSS after mini-open carpal tunnel release and baseline BCTQ-SSS score. Black circles represent values of individual studies where the circle size is proportional to the study weight in the random-effects model. The red line represents the regression line of best fit with the shaded area indicating the 95% confidence interval. The regression equation to predict BCTQ-SSS change = [1.63 – (1.09 * baseline BCTQ-SSS)]; p<0.001. BCTQ-SSS, Boston Carpal Tunnel Questionnaire Symptom Severity Scale.

**Supplement Figure 5.** Bubble plot of the association between the change in BCTQ-FSS after mini-open carpal tunnel release and baseline BCTQ-FSS score. Black circles represent values of individual studies where the circle size is proportional to the study weight in the random-effects model. The red line represents the regression line of best fit with the shaded area indicating the 95% confidence interval. The regression equation to predict BCTQ-FSS change = [1.27 – (0.98 * baseline BCTQ-FSS)]; p<0.001. BCTQ-FSS, Boston Carpal Tunnel Questionnaire Functional Status Scale.

**Supplement Figure 6.** Bubble plot of the association between the change in pain VAS after mini-open carpal tunnel release and baseline pain VAS score. Black circles represent values of individual studies where the circle size is proportional to the study weight in the random-effects model. The red line represents the regression line of best fit with the shaded area indicating the 95% confidence interval. The regression equation to predict pain VAS change = [-1.1 – (0.7 * baseline pain VAS)]; p<0.001. VAS, visual analogue scale.


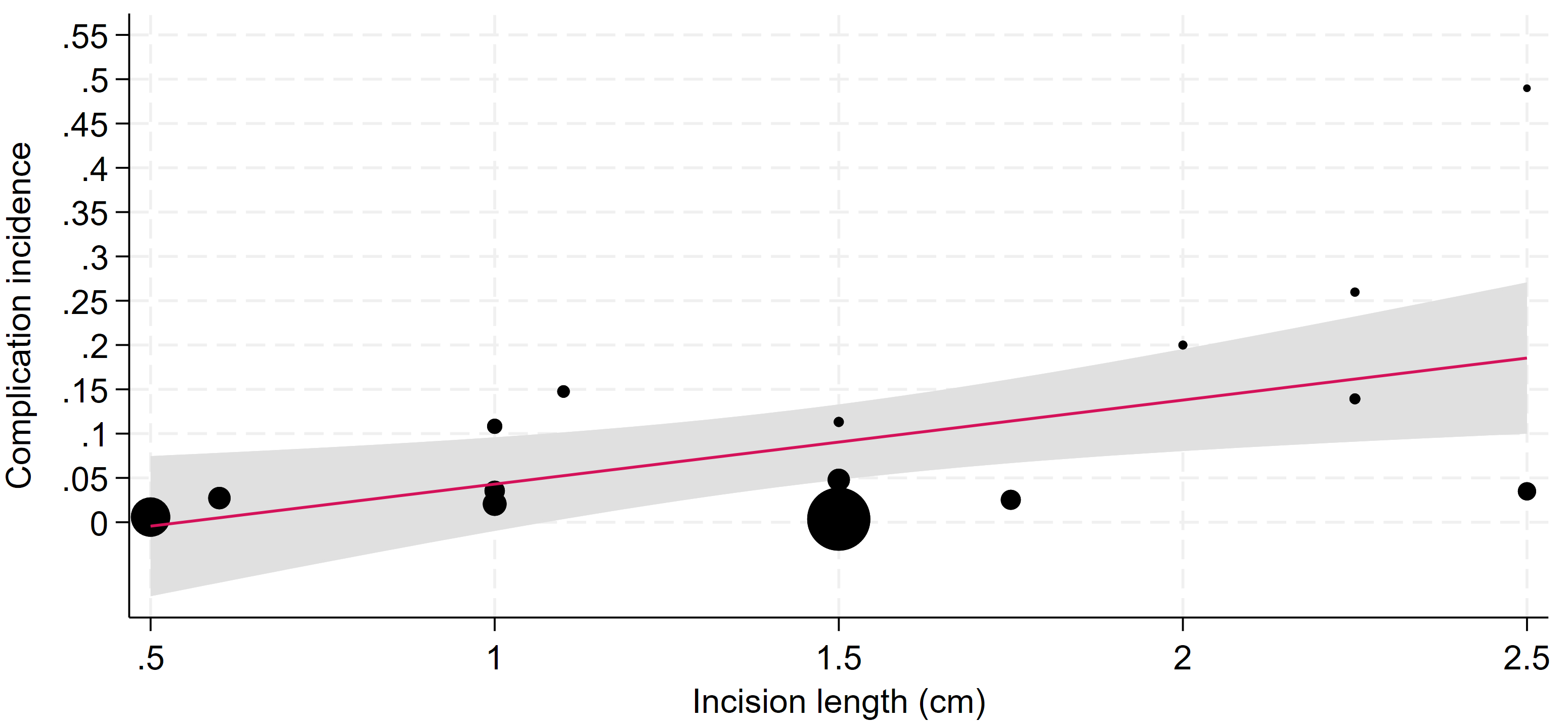


**Supplement Figure 7.** Bubble plot of the association between the incidence of complications after mini-open carpal tunnel release and surgical incision length. Black circles represent values of individual studies where the circle size is proportional to the study weight in the random-effects model. The red line represents the regression line of best fit with the shaded area indicating the 95% confidence interval. The regression equation to predict complication incidence = [-0.052 + (0.095 * incision length)]; p<0.001.
